# Porphyromonas gingivalis associates with the presence of anti-citrullinated protein antibodies, but not with the onset of arthritis: studies in an at-risk population

**DOI:** 10.1136/rmdopen-2024-005111

**Published:** 2025-01-31

**Authors:** Charlotte De Vries, Alexandra Cîrciumaru, Linda Mathsson-Alm, Helga Westerlind, Marina Dehara, Yogan Kisten, Barbara Potempa, Jan Potempa, Aase Hensvold, Karin Lundberg

**Affiliations:** 1 Division of Rheumatology, Department of Medicine Solna, Karolinska Institutet and Center for Molecular Medicine, Karolinska University Hospital, Stockholm, Sweden; 2 Center for Rheumatology, Academic Specialist Center, Stockholm Health Services, Region Stockholm, Stockholm, Sweden; 3 Department of Immunology, Genetics and Pathology, Uppsala University, Uppsala, Sweden; 4 Thermo Fisher Scientific, ImmunoDiagnostics Division, Uppsala, Sweden; 5 Clinical Epidemiology Division, Department of Medicine Solna, Karolinska Institutet, Stockholm, Sweden; 6 Department of Oral Immunology & Infectious Diseases, School of Dentistry, University of Louisville, Louisville, Kentucky, USA; 7 Faculty of Biochemistry, Biophysics and Biotechnology, Jagiellonian University, Krakow, Poland

**Keywords:** Anti-Citrullinated Protein Antibodies, Arthritis, Rheumatoid, Epidemiology, Risk Factors, Biomarkers

## Abstract

**Objective:**

The antibody response against *Porphyromonas gingivalis* (*Pg*) is elevated in rheumatoid arthritis (RA), especially in patients with anti-citrullinated protein antibodies (ACPA). Here, we investigated whether antibodies against the *Pg* virulence factor arginine gingipain (Rgp) are associated with the RA-risk phase and development of arthritis.

**Methods:**

At-risk individuals were included in a prospective study (Risk-RA) based on a positive anti-cyclic citrullinated peptide 2 (CCP2) antibody test, and having musculoskeletal complaints but no signs of arthritis. Study participants were followed for ≥3 years (arthritis-free, n=165) or until arthritis onset (progressors, n=95). Anti-Rgp IgG was measured in Risk-RA (260 baseline and 247 follow-up samples) and healthy controls (n=126); anti-CCP2 IgG was measured in Risk-RA (254 baseline samples). Data were analysed in GraphPad Prism and R using log-transformed antibody levels.

**Results:**

53% of Risk-RA and 26% of controls, p=0.003, were anti-Rgp IgG positive at baseline, with higher levels in Risk-RA compared with controls, p<0.0001. No changes in anti-Rgp IgG levels were observed during follow-up. The anti-Rgp IgG response at baseline did not associate with the development of arthritis; Cox-regression showed an HR of 0.95 (CI 0.80 to 1.13, p=0.6) for anti-Rgp IgG levels, and 0.82 (CI 0.55 to 1.23, p=0.3) for anti-Rgp IgG positivity.

**Conclusions:**

Antibodies against the oral bacterium *Pg* are elevated during the RA-risk phase, both in individuals progressing to arthritis and in individuals remaining arthritis-free. Hence, *Pg* infection can be linked to the presence of RA-specific autoimmunity, ACPA, and musculoskeletal symptoms, but not to further development of arthritis in this at-risk population.

**WHAT IS ALREADY KNOWN ON THIS TOPIC**

- Several studies support an aetiological link between periodontal infection/-inflammation by *Porphyromonas gingivalis* (*Pg*) and anti-citrullinated protein antibody (ACPA)-positive rheumatoid arthritis (RA). However, studies aimed at differentiating the possible impact of this risk factor on systemic autoimmunity versus arthritis are limited and inconclusive.

**WHAT THIS STUDY ADDS**

- This study shows that the antibody response to *Pg* virulence factor arginine gingipain (Rgp)—as a proxy for *Pg* infection—associates with the risk phase of RA, here defined by the presence of ACPA and musculoskeletal complaints, but not to further risk of developing arthritis.
- The data presented here links *Pg* to the ‘first hit’ in the ‘two-hit’ aetiological model for ACPA-positive RA, that is, the break of tolerance and systemic autoimmunity, preceding the development of arthritis.

**HOW THIS STUDY MIGHT AFFECT RESEARCH, PRACTICE OR POLICY**

- This study can contribute to an increased understanding of disease mechanisms underpinning ACPA-positive RA and highlights the importance of oral health and preventive measures concerning a modifiable risk factor.

## Introduction

 Studies demonstrate an increased prevalence of periodontitis among rheumatoid arthritis (RA) patients.^1^ A recent study also suggests a link between oral bacteraemia and RA flares,^2^ highlighting the role of oral health in RA. *Porphyromonas gingivalis* (*Pg*), a key pathogen driving periodontitis, has specifically been implicated in contributing to the development of anti-citrullinated protein antibody (ACPA)-positive RA due to its capacity to citrullinate proteins by expression of a peptidyl arginine deiminase (PAD) enzyme,^3 4^ and by triggering release of neutrophil extracellular traps containing citrullinated histones.^5^ Studies also show that RA patients, in particular ACPA-positive, have higher anti-*Pg* antibody levels compared with controls,^6 7^ and a higher relative abundance of *Pg* was described in ACPA-positive at-risk individuals compared with controls.^8^ In retrospective studies, we have previously seen elevated antibody levels against arginine gingipain (Rgp), an important *Pg* virulence factor, in RA versus controls, even before RA symptom onset,^6 9 10^ and we have observed an interaction between anti-Rgp IgG and *HLA-DRB1* shared epitope (SE) in ACPA-positive RA.^6^

In the context of the hypothesis that genetic and environmental risk factors together contribute to (1) break of tolerance and systemic autoimmunity followed by (2) onset of arthritis, in a ‘two-hit model’, investigation of the risk-phase of RA is particularly relevant. Better knowledge of risk factors acting at different stages prior to disease is important for an increased understanding of disease mechanisms and preventive measures. Hence, to differentiate the possible effects of *Pg* on the risk phase versus arthritis phase, we have analysed anti-Rgp IgG in a large prospective ACPA-positive at-risk cohort, where 37% developed arthritis during the study period.

## Methods

### Study population

We included 260 individuals from the Karolinska Risk-RA prospective cohort.^11^ Inclusion criteria were a positive anti-cyclic citrullinated peptide 2 (CCP2) antibody test (performed in primary care) and musculoskeletal complaints (MSK-C) with no prior/current rheumatic disease or signs of clinical/ultrasound-detected arthritis (assessed at the rheumatology clinic). Participants had annual follow-up visits for ≥3 years (n=165 arthritis-free) or until clinical arthritis developed (n=95 progressors). Serum was available from study baseline (n=260), ≥1 intermediate follow-up timepoint (n=44 progressors, n=92 arthritis-free), and arthritis onset (n=48 progressors) or study endpoint (n=37 arthritis-free). Baseline characteristics are described in table 1.

**Table 1:** Baseline characteristics of ACPA-positive Risk-RA individuals

	Risk-RA (all) N=260	Arthritis-free N=165	Progressors N=95	P value
Female sex, n (%)	204 (78)	127 (77)	77 (81)	0.5
Age, years, mean (SD)	48 (14)	47 (15)	50 (13)	0.06
Time from inclusion to last follow-up, months, median (IQR)	49 (22–59)	54 (49–68)	13 (6–27)	**<0.0001**
Current smoker, n (%)	36 (15)	19 (12)	17 (19)	**0.04**
RF positive*, n (%)	85 (33)	37 (22)	48 (51)	**<0.0001**
*HLA*-SE positive†, n (%)	156 (60)	89 (54)	67 (71)	**0.009**
CCP2 IgG levels‡, median (IQR)	10 (3–78)	6 (2–27)	50 (9–100)	**<0.0001**

Significant p-values are shown in bold.

* Information on RF status was retrieved from primary care (routine clinical laboratory);.

† HLA genotyping was performed previously.^11^ ;

‡ CCP2 IgG levels were retrieved from primary care (routine clinical laboratory) and normalizednormalised to cut-off.

CCP2, cyclic citrullinated peptide 2*HLA*-SE, human leucocyte antigen-shared epitope; RF, rheumatoid factor

Healthy controls (HC) (n=126) were matched to Risk-RA based on sample handling and sex (79% women). Median age was 48 years for Risk-RA and 54 years for HC, p=0.004. Information on periodontal status was not available.

## ELISA

Sera from Risk-RA (n=507 samples from 260 individuals) and HC (n=126) were analysed, using a previously described *in-house* ELISA, for the presence of anti-Rgp IgG, using recombinant RgpB protein^6^ (online supplemental methods and online supplemental figure 1). Risk-RA baseline samples (n=254) were additionally analysed for anti-CCP2 IgG, using the EliA CCP2 assay, Phadia AB part of Thermo Fisher Scientific.

### Statistics

Analyses were performed using R, V.4.3.1, and GraphPad Prism, V.10.0.3. T-test, Mann-Whitney U test, χ^2^ test (group comparisons), analysis of variance (longitudinal samples) and Spearman test (correlation) were applied to natural log-transformed anti-Rgp IgG data. Anti-Rgp IgG cut-off was calculated using Youden’s J statistic based on receiver operating characteristics (ROC) curve analysis. Cox proportional hazard univariate/multivariate models were applied using log-transformed anti-Rgp IgG levels/positivity; sensitivity analyses included age, sex, smoking status, interleukin (IL)-6, IL-15 receptor (R)α, tenosynovitis and presence of any ACPA fine-specificity.^11^ P values <0.05 were considered statistically significant.

## Results

### Anti-Rgp IgG at baseline

**Figure 1:**
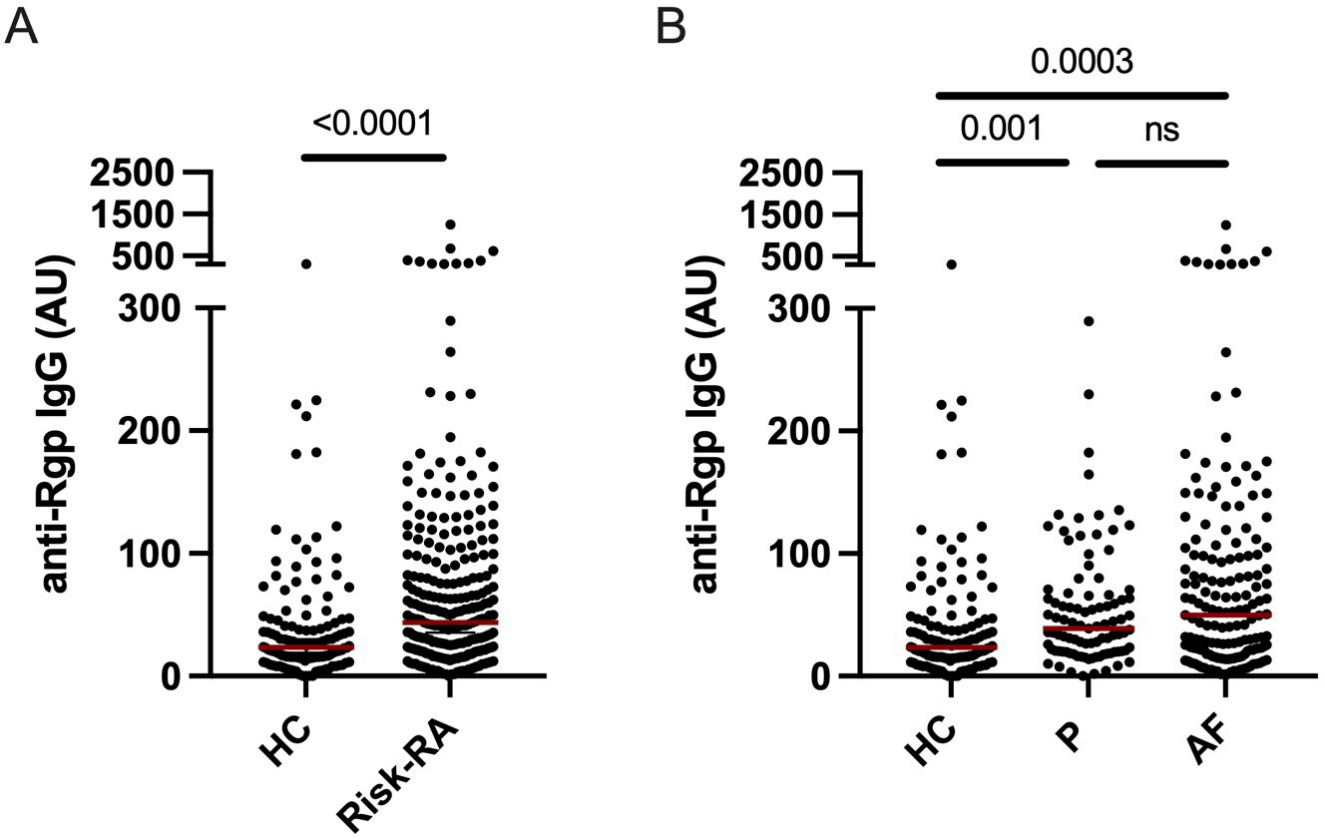
Baseline anti-Rgp IgG levels are elevated in Risk-RA individuals. Graphs show baseline anti-Rgp IgG levels in Risk-RA individuals (n=260) and healthy controls (n=126) (**A**), as well as in healthy controls, Risk-RA progressors (n=95) and Risk-RA arthritis-free individuals (n=165) (**B**); red horizontal lines depict median values. AF, arthritis free; AU, arbitrary units; HC, healthy controls; ns, non-significant; P, progressors; Rgp, arginine gingipain.

We detected increased baseline levels of anti-Rgp IgG in Risk-RA (median: 44 arbitrary units (AU), IQR: 20–91) compared with controls (median: 24 AU, IQR: 15–46), p<0.0001 (figure 1A), with no statistically significant difference between progressors (median: 39 AU, IQR: 20–66) and arthritis-free (median: 50 AU, IQR: 20–100), p=0.34 (figure 1B). Both progressors (p=0.001) and arthritis-free (p=0.0003) had higher levels than controls.

Applying ROC curve analysis, anti-Rgp IgG levels could separate Risk-RA individuals from controls with an area under the curve of 0.64 (CI 0.58 to 0.70), giving a sensitivity of 53% (CI 47 to 59) and specificity of 74% (CI 66 to 81) (online supplemental figure 2). Using this cut-off, 48% of progressors and 55% of arthritis-free were positive for anti-Rgp IgG at baseline, p=0.4.

As previously shown, high baseline anti-CCP2 antibody levels, presence of rheumatoid factor (RF) and carriage of *HLA-DRB1* SE are associated with progression to arthritis (table 1).^11^ Anti-Rgp IgG showed no association with these factors, but was associated with male sex, younger age and current smoking (online supplemental table 1).

### Longitudinal investigation of anti-Rgp IgG levels

**Figure 2:**
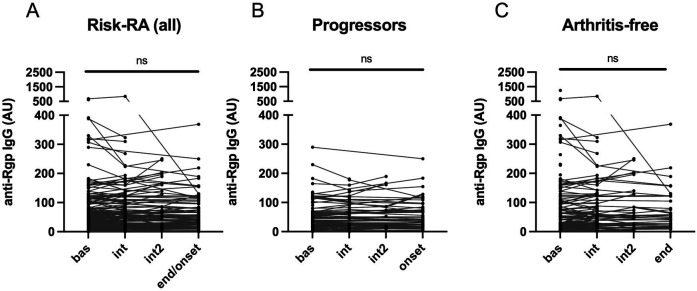
Anti-Rgp IgG levels remain stable during the Risk-RA phase. Graphs show anti-Rgp IgG levels over time, in all Risk-RA individuals with ≥1 follow-up sample available (bas: n=184, int: n=136, int2: n=30, end/onset: n=80) (**A**), all Risk-RA individuals divided into progressors (bas: n=95, int: n=44, int2: n=9, onset: n=48) (**B**) and arthritis-free (bas: n=165, int: n=92, int2: n=21, end: n=33) (**C**). AU, arbitrary units; bas, baseline; end, study endpoint; int/int2, intermediate time points; ns, non-significant; onset, time of arthritis onset; Rgp, arginine gingipain.

We detected stable anti-Rgp IgG levels over time in Risk-RA, also when separating progressors from arthritis-free (figure 2). The frequency of anti-Rgp IgG positive individuals did not alter significantly over time, and anti-Rgp IgG levels/-positivity did not differ between progressors and arthritis-free at any timepoint (data not shown).

### Anti-Rgp IgG in relation to arthritis onset

34% of anti-Rgp IgG positive individuals, and 40% of anti-Rgp IgG negative, progressed to arthritis, p=0.4 (online supplemental table 1). Univariate Cox regression, accounting for follow-up time, showed no statistically significant association of anti-Rgp IgG levels to the onset of arthritis (HR: 0.95, CI 0.80 to 1.13, p=0.6), nor did anti-Rgp IgG positivity (HR: 0.82, CI 0.55 to 1.23, p=0.3); sensitivity analysis, including sex, age and smoking status, did not alter outcomes, nor did additional sensitivity analysis, including factors recently described as strong predictors of arthritis onset in this at-risk cohort, that is, IL-6, IL-15Rα, tenosynovitis at baseline and presence of any ACPA fine-specificity^11^ (online supplemental table 2).

### ACPA seroconversion in relation to anti-Rgp IgG and arthritis onset

Despite having a positive CCP2 test in primary care, re-analysis of Risk-RA individuals at the study baseline revealed that 38% had seroconverted, that is, lost CCP2-reactivity. Seroconverters were largely borderline CCP2-positive in primary care, younger than non-converters, p=0.001 (online supplemental figure 3A,B), and only 8%, versus 54% of non-converters, progressed to arthritis, p<0.001 (data not shown). Anti-Rgp IgG levels did not differ between CCP2-converters and non-converters (online supplemental figure 3C), or associate with arthritis progression, CCP2 IgG levels, RF, *HLA-DRB1* SE, age, sex and smoking, when only Risk-RA individuals who remained CCP2-positive at study baseline were analysed (online supplemental table 3).

## Discussion

We show that the anti-Rgp antibody response is elevated in ACPA-positive at-risk individuals with MSK-C compared with HC, with stable levels during the risk phase, in both progressors and those who remained arthritis-free during the study period.

Our findings are in line with other reports, where anti-*Pg* antibodies could not predict progression to RA.^10 12 13^ Two of these studies used *Pg* lysates^12^ or *Pg* outer membrane proteins^13^ to capture anti-*Pg* antibodies, which may contain citrullinated epitopes due to citrullination by *P*.PAD,^14^ leading to false-positive anti-*Pg* antibody detection in ACPA-positive individuals. Our study, like Fisher *et al*,^10^ used purified gingipain, previously suggested to be ideal for *Pg* screening,^15^ excluding the risk of *P.*PAD contamination. Moreover, our study, like de Smit *et al*,^12^ compared prospectively collected samples, while the other studies were retrospective, and lacked information on MSK-C before RA diagnosis.^10 13^ Additionally, our study comprised only CCP2-positive individuals, while both CCP2-positive and CCP2-negative individuals were included in the other studies.

The fact that we found elevated anti-Rgp IgG levels in ACPA-positive at-risk individuals compared with ACPA-negative controls, and that levels remained stable over time, did not predict the onset of arthritis, or associate with factors known to predict arthritis onset in this Risk-RA cohort (eg, high anti-CCP2 IgG levels, RF and *HLA-DRB1* SE^11^), suggest that anti-Rgp IgG could be related to the autoimmune ACPA-response per se rather than to arthritis. Hence, our data support a model whereby *Pg* infection is linked to the ‘first hit’ in the cascade of events leading up to ACPA-positive RA.

Since the presence of anti-Rgp antibodies reflects *Pg* infection, the data could implicate that *Pg* infection triggers ACPA-production, as proposed by Rosenstein *et al.*^3^ We know from our earlier retrospective study, that elevated anti-Rgp IgG levels are long-term stable during 10–12 years before RA diagnosis, while ACPA levels gradually increase during this time,^9^ suggesting that *Pg* infection may precede the ACPA-response. Furthermore, we have recently shown an association between elevated anti-Rgp IgG levels and a high degree of gingival inflammation in periodontitis,^16^ and since gingival inflammation associates with extensive citrullination in a pathobiont-rich milieu,^17^ we speculate that this could facilitate break of tolerance to citrullinated proteins. In support of this scenario, ACPA has been detected in gingival crevicular fluid, and we have identified citrulline-reactive gingival tissue B cells.^18 19^

A limitation of the study is that we cannot say with certainty that those who remained arthritis-free within the study period will not progress in the future.

The observation that 38% of CCP2-positive Risk-RA individuals with MSK-C seroconverted between the first visit in primary care and the study baseline visit, in line with a previous report,^20^ needs further investigation and longer follow-up time. Potentially, these (significantly younger) individuals were identified at an earlier stage, when CCP2 IgG levels may fluctuate around cut-off, before the ACPA response matures.

In conclusion, anti-Rgp IgG as a proxy for *Pg* infection can be linked to the risk phase of ACPA-positive RA but does not further predict arthritis onset, at least not within the timeframe of this study.

## supplementary material

10.1136/rmdopen-2024-005111 online supplemental file 1

## Data Availability

Data is available upon reasonable request.
