# Supplementary material for: Porphyromonas gingivalis associates with the presence of anti-citrullinated protein antibodies, but not with the onset of arthritis: studies in an at-risk population
Source: RMD Open. 2025 Jan 31;11(1):e005111. doi: 10.1136/rmdopen-2024-005111 (PMC11792289; doi:10.1136/rmdopen-2024-005111)
Supplement: online supplemental file 1 [file rmdopen-11-1-s001.docx]

**SUPPLEMENTARY MATERIAL**

**SUPPLEMENTARY METHODS**

**Anti-Rgp IgG ELISA**

Nunc MaxiSorp 96-well half-area ELISA plates were coated over night at 4°C with recombinant RgpB protein (1) (50µl/well) at a concentration of 2.8µg/mL (diluted in 50mM carbonate buffer, pH 9.5). Plates were subsequently washed (x3 in phosphate buffered saline (PBS); 0.05% Tween) and blocked (PBS; 2% bovine serum albumin, 100µl/well) for 2h at room temperature (RT). Serum samples, diluted 1:400 in RIA buffer (1% (w/v) BSA, 350mM NaCl, 10mM Tris-HCl (pH 7.6), 1% (v/v) Triton X-100, 0.5% (w/v) Na-deoxycholate, 0.1% (w/v) SDS), were added in duplicates (50µl/well) and incubated for 1.5h at RT. Plates were washed again and incubated with peroxidase-conjugated mouse anti-human IgG (50µl/well; diluted 1:10 000 in RIA buffer) for 1.5h at RT. Following a final wash, antibody binding was detected using tetramethylbenzidine substrate (50µl/well); the reaction was stopped after 15-18 minutes by adding 1M H_2_SO_4_ (50µl/well) and optical density (OD) measured at 450nm in a SpectraMax Plus 384 microplate reader.

To compare serum samples analyzed on different ELISA plates, antibody levels were presented as arbitrary units (AU), calculated from a standard curve included on all plates (see supplementary figure 1). The standard curve was based on a pool of anti-Rgp IgG-positive sera added in serial dilution (1:1 dilutions x7). Blank wells were included on all plates to account for background signal, with the blank OD subtracted from the OD values of the standard curve and samples.

Youden's J statistic was applied to the receiver operating characteristic (ROC) curve analysis (n=260 Risk-RA; n=126 healthy controls) to identify the anti-Rgp IgG cut-off value (40.9 AU) with the highest combined sensitivity and specificity.

**SUPPLEMENTARY FIGURES**

**

**Supplementary figure 1.** The graph shows a representative anti-Rgp IgG standard curve, with AU values on the X-axis, plotted using natural log scale, and corresponding OD values on the Y-axis. The red line depicts cut-off for anti-Rgp IgG positivity, determined using Youden’s J statistic, derived from receiver operating characteristic (ROC) curve analysis of n=260 Risk-RA individuals and n=126 healthy controls. AU = arbitrary units; IgG = immunoglobulin G; OD = optical density; Rgp = arginine gingipain.

**Supplementary figure 2**. **Anti-Rgp IgG separate Risk-RA individuals from healthy controls**. Graphs show the receiver operating characteristic (ROC) curve of baseline anti-Rgp IgG levels in 260 Risk-RA individuals and 126 healthy controls (A), and the distribution of anti-Rgp antibody positive and anti-Rgp antibody negative Risk-RA individuals and healthy controls (B). HC = healthy controls; Rgp = arginine gingipain.

**Supplementary figure 3**. **Antibody levels and age in CCP2-converters and non-converters**. Graphs show anti-CCP2 IgG levels from primary care, normalized to cut-off (A), age (B), and study baseline anti-Rgp IgG levels (C), in Risk-RA individuals positive (n=158) or negative (n=96) for anti-CCP2 IgG at study baseline. AU = arbitrary units; CCP2 = cyclic citrullinated peptide2; IgG = immunoglobulin G; Rgp = arginine gingipain.

**SUPPLEMENTARY TABLES**

**Supplementary table 1**. **Characteristics of ACPA-positive Risk-RA individuals, divided by anti-Rgp IgG status**

|  | Rgp IgG+  N=136 | Rgp IgG-  N=124 | P value |
| --- | --- | --- | --- |
| Progression to arthritis, n (%) | 46 (34) | 49 (40) | 0.4 |
| Female sex, n (%) | 100 (74) | 104 (84) | 0.049 |
| Age, years, mean (SD) | 46 (14) | 50 (14) | 0.02 |
| Time from inclusion to last follow-up, months, median (IQR) | 49 (27-59) | 48 (16-60) | 0.7 |
| Current smoker, n (%) | 25 (18) | 11 (9) | 0.046 |
| RF positive*, n (%) | 46 (34) | 39 (31) | 0.7 |
| HLA-SE positive**, n (%) | 81 (60) | 75 (60) | 0.99 |
| CCP2 IgG levels***, median (IQR) | 8 (3-71) | 12 (3-90) | 0.4 |

*Information on RF status was retrieved from primary care (routine clinical laboratory); **HLA genotyping was performed previously (2); ***CCP2 IgG levels were retrieved from primary care (routine clinical laboratory) and normalized to cut-off. CCP2 = cyclic citrullinated peptide2; HLA-SE = human leukocyte antigen – shared epitope; IgG = immunoglobulin G; IQR = inter quartile range; N = number; RF = rheumatoid factor; Rgp = arginine gingipain; SD = standard deviation.

**Supplementary table 2**. **Summary of Cox regression analysis**

| Outcome | Variables | HR (Rgp) | CI | P value |
| --- | --- | --- | --- | --- |
| Arthritis onset | Anti-Rgp IgG levels | 0.95 | 0.80 -1.13 | 0.6 |
| Arthritis onset | Anti-Rgp IgG (binary) | 0.82 | 0.55-1.23 | 0.3 |
| Arthritis onset | Anti-Rgp IgG levels, sex, age, current smoking | 0.94 | 0.79-1.13 | 0.5 |
| Arthritis onset | Anti-Rgp IgG (binary), sex, age, current smoking | 0.84 | 0.55-1.23 | 0.4 |
| Arthritis onset | Anti-Rgp IgG levels, IL-6, IL-15Rα*, tenosynovitis, ACPA** | 0.90 | 0.75-1.08 | 0.2 |
| Arthritis onset | Anti-Rgp IgG (binary), IL-6, IL-15R*, tenosynovitis, ACPA** | 0.75 | 0.49-1.13 | 0.2 |

*IL-6 and IL-15Rα levels, previously analysed on Olink® multiplex (2); **presence of any ACPA fine-specificity, previously analysed on a custom-made multiplex microarray (2). ACPA = anti-citrullinated protein antibody; CI = confidence interval; HR = hazard ratio; IgG = immunoglobulin G; IL = interleukin; R = receptor; Rgp = arginine gingipain.

**Supplementary table 3**. **Characteristics of ACPA-positive Risk-RA individuals** (CCP2-positive at study baseline)**,** **divided by anti-Rgp IgG status**

|  | Rgp IgG+  N=76 | Rgp IgG-  N=82 | P value |
| --- | --- | --- | --- |
| Progression to arthritis, n (%) | 40 (53) | 45 (58) | 0.5 |
| Female sex, n (%) | 57 (75) | 68 (84) | 0.2 |
| Age, years, mean (SD) | 50 (14) | 52 (13) | 0.5 |
| Time from inclusion to last follow-up, months, median (IQR) | 40 (13-56) | 41 (12-67) | 0.7 |
| Current smoker, n (%) | 16 (22) | 10 (13) | 0.2 |
| RF positive*, n (%) | 51 (67) | 56 (68) | 0.99 |
| HLA-SE positive**, n (%) | 48 (66) | 52 (66) | 0.99 |
| CCP2 IgG levels***, median (IQR) | 277 (72-600) | 144 (55-493) | 0.13 |

*Information on RF status was retrieved from primary care (routine clinical laboratory); **HLA genotyping was performed previously (2); ***CCP2 IgG levels measured at study baseline, presented in arbitrary units. CCP2 = cyclic citrullinated peptide2; HLA-SE = human leukocyte antigen – shared epitope; IgG = immunoglobulin G; IQR = inter quartile range; N = number; RF = rheumatoid factor; Rgp = arginine gingipain; SD = standard deviation.

**REFERENCES**

1. Veillard F, Potempa B, Guo Y, Ksiazek M, Sztukowska MN, Houston JA, et al. Purification and

characterisation of recombinant his-tagged rgpb gingipain from porphymonas gingivalis. Biol

Chem. 2015;396(4):377-384.

2. Cîrciumaru A, Kisten Y, Hansson M, Mathsson-Alm L, Joshua V, Wähämaa H, et al. Identification of early risk factors for anti-citrullinated-protein-antibody positive rheumatoid arthritis - a prospective cohort study. Rheumatology (Oxford). 2024;8:keae146.
